# QiLing Decoction promotes ferroptosis of castration-resistant prostate cancer cells by inhibiting FSP1 *in vitro* and *in vivo*

**DOI:** 10.7150/jca.84363

**Published:** 2023-07-16

**Authors:** Hongwen Cao, Xiaotong Wu, Renjie Gao, Lei Chen, Yigeng Feng, Dan Wang

**Affiliations:** Surgical Department I (Urology Department), LONGHUA Hospital Shanghai University of Traditional Chinese Medicine, No. 725 Wanping Road South, Xuhui District, Shanghai 200032, China.

**Keywords:** Qiling Decoction, prostate cancer, castration-resistant prostate cancer, ferroptosis, FSP1

## Abstract

QiLing Decoction (QLD) showed therapeutic effects against prostate cancer with an unclear underlying mechanism. This study explored the underlying mechanisms of QLD against castration-resistant prostate cancer (CRPC). Clinical specimens were collected from the patients with CRPC. Stable cells including knockdown and overexpression cell lines were established by plasmid transfection. The xenograft animal model was constructed. Cell viability was determined by using cell-counting kit 8 assay. Biochemical assays were used to determine the levels of iron (Fe^2+^) and lipid reactive oxygen species (ROS). qRT-PCR and Western blotting were used to determine levels of target genes, respectively. Treatment of QLD inhibited ferroptosis suppressor protein (FSP) 1 at mRNA and protein levels in patients with CRPC. Additionally, cells treated with QLD-containing serum displayed a decrease in cell viability and an increase in Fe^2+^ and lipid ROS with or without erastin, whereas ferroptosis inhibitor reversed QLD-induced ferroptosis. The regulatory effects of QLD on PC3 cell ferroptosis were associated with its inhibitory effects against FSP1. Consistently, QLD inhibited PC3 tumor growth by inhibiting FSP1. Moreover, treatment of QLD increased the sensitivity of PC3-AbiR cells to abiraterone by inhibiting FSP1. QLD promoted ferroptosis in CRPC cells in part by inhibiting FSP1 *in vitro* and *in vivo*.

## Introduction

Prostate cancer is the second most common cancer type and the fifth leading cause of cancer-related death among male cancers 1. Besides, it is the fourth most common cancer among all cancer types with more than 1,400,000 diagnosed cases globally in 2020 1. Several factors, including hormones, race, family history, diet, chemicals, and aging, raise the risk of developing prostate cancer 2. And around 60%~ of prostate cancer cases are diagnosed in patients aged 65 and older 2. Most of the patients with prostate cancer were diagnosed in the early stage, which is highly curable (5-year relative survival rate > 99%) 3. However, once cancer spread and metastasis into the distant areas, 5-year relative survival rate drops to 31% 3. Hormone therapy, also called androgen deprivation therapy (ADT), is one of the standard therapeutic options for the treatment of advanced prostate cancer by reducing or blocking the stimulation effects of the hormone on cancer cell growth 4. ADT has proven to be one of the most effective therapies against patients with hormone-sensitive prostate cancer 4. The previous study demonstrated ADT's effectiveness on at least 40% of patients aged 65 and older with prostate cancer without accepting other therapeutic options 5. However, ADT therapy in long term can result in the development of castration-resistant prostate cancer (CRPC) 6. Several antiandrogen medications, such as enzalutamide, apalutamide, and abiraterone, have been introduced in the treatment of CRPC 7. Antiandrogen drug resistance is one of the major risks for those antiandrogen medications 6. Therefore, it is urgently needed to find effective therapeutic options against CRPC.

Ferroptosis is a newly discovered programmed cell death dependent on iron. The occurrence of ferroptosis is accompanied by the accumulation of irons (Fe^2+^) and lipid peroxides 8. It is associated with many types of cancers including hepatoma, melanoma, colorectal cancer, prostate cancer, etc. 9,10, More recently, several studies suggested that the regulation of ferroptosis is one of the novel therapeutic approaches against advanced prostate cancer 11,12. Interestingly, in 2022, Chen and colleagues reported that inducing ferroptosis can improve docetaxel's sensitivity in prostate cancer cells, suggesting the association between ferroptosis and prostate cancer drug resistance 13. Therefore, it is interesting to explore the relationship between ferroptosis and antiandrogen drug resistance in prostate cancer.

QiLing decoction (QLD) is a traditional Chinese medicine (TCM), which displays medicinal properties against advanced prostate cancer 14-16. In 2021, our group reported that QLD suppressed docetaxel resistance and aerobic glycolysis in patients with CRPC, at least in part, by the regulation of long non-coding RNA SNHG10 14. In 2022, their bio-informatic study further suggested a total of 51 active components and 149 potential bio-targets for QLD in the treatment of CRPC 15. However, it is still unclear if QLD can also improve the sensitivity of the antiandrogen drug against prostate cancer cells and their underlying mechanisms. In this study, we aimed to explore the roles of QLD in the CRPC. Moreover, we investigated the effects of QLD on antiandrogen drug resistance in the CRPC and its molecular mechanisms.

## Material and Method

### Chemicals and primary antibodies

Erastin, liproxstatin-1, and abiraterone (Abi) were obtained from the MedChemExpress (MCE, Monmouth Junction, NJ, USA). Qi Ling Decoction (QDL) consists of the Traditional Chinese Medical (TCM) components including kushan (150 g), rubescens (300 g), raw astragalus (150 g), turmeric (90 g), psoraleae (150 g), cooked rehmannia glutinosa (150 g), motherwort (150 g), and processed licorice (90 g) as previously reported 16. These TCM components were mixed and boiled in hot water for 4 hours to make QDL solution. Drug-Containing Serum of QDL was prepared as follows. Male Lewis rat (180-220 g) were orally administrated with QDL solution at a dosage of 10 ml/kg twice a day for continuous 7 days. On day 7, after administration of QDL solution for 1 hour, blood was collected from the abdominal aortic. After centrifugation for 15 mins, the serum was separated and then inactivated at 56°C for 30 mins. Next, the serum was kept at -80°C after filtering with 0.22 µm for further use.

The primary antibody against GADPH was purchased from Sigma-Aldrich (St. Louis, MO). Primary antibody against Ferroptosis Suppressor Protein (FSP) 1 or Solute Carrier Family 7 Member (SLC7A) 11 was purchased from Cell Signaling Technology (CST, Danvers, MA). Primary antibody against Solute Carrier Family 3 Member (SLC3A) 2 or Glutathione peroxidase (GPX) 4 was purchased from Thermo Fisher Scientific (Waltham, MA).

### Cell culture

Cell lines PC3, DU145 and RWPE-1 were obtained from the American Type Culture Collection (Manassas, VA, USA). PC3 and DU145 were maintained in the RPMI-1640 medium (Gibco, Grand Island, NY) with 10% fetal bovine serum (FBS) in the presence of 1% penicillin/streptomycin solution (Gibco). The abiraterone-resistant PC3 cell line was constructed by incubating monolayer-cultured cells with Abi at incremental concentrations as previously reported. RWPE-1 cells were cultured in keratinocyte serum free media supplemented with bovine pituitary extract (0.05 mg/ml) and epidermal growth factor (5 ng/ml). PC3, DU145 and RWPE-1 were cultured in a cell culture incubator at 37°C in a humidified atmosphere with 5% CO_2_.

### Animals and experimental design

BALB/c nude mice were purchased from Shanghai Model Organisms (Shanghai, China) with free access to chow diet and water. Two cohorts of animal study were designed in this current study as described follows.

To establish the xenograft tumor model, mice were subcutaneously injected with 5×10^6^ tumor cells. Twenty mice were divided into four groups with a group of five mice. For the control and QLD groups, the mice were subcutaneously injected with 5×10^6^ PC3 cells. For the shControl and shFSP1 groups, the mice were subcutaneously injected with 5×10^6^ shControl- or shFSP1-transfected PC3 cells. In the control group, the mice were gavaged with water. In the QLD group, the mice were gavaged with QLD. In the shControl and shFSP1 groups, the mice were gavaged with water. In another cohort study, twenty mice were divided into four groups with a group of five mice. For the shControl group and shControl plus Abi, the mice were subcutaneously injected with 5×10^6^ shControl-transfected PC3 cells and shControl-transfected PC3 cells incubated with Abi, respectively. For the QLD plus Abi and shFSP1 plus Abi groups, the mice were subcutaneously injected with 5*10^6^ QLD-treated and shFSP1-transfected PC3 cells, respectively. Tumor volume was measured every seven days according to the formula: V = 0.5 × a × b^2^ (a: length of tumor; b: short diameter of tumor). Tumor tissues were collected at the end of the study and kept for further use.

### Clinical specimen

Patients with CRPC (n = 13) enrolled in this study were recruited. Eligible patients had a prostate cancer and disease progression on ADT (serum testosterone ≤ 1.73 nM), as per the Prostate Cancer Working Group 3 criteria. Inclusion criteria included 18-90 years old, Eastern Cooperative Oncology Group (ECOG) performance score of 0-1, normal liver, kidney and cardiac function. Exclusion criteria included previous (in one year) or current evidence of infectious disease, cardiovascular disease, diabetes mellitus, neurological disease, and other malignant tumors assessed by magnetic resonance imaging or computed tomography. All patients have read and signed the content of the form. The tumor biopsy specimen was collected from these patients before and after 2-month treatment of QLD. The study was approved by the ethics committee LONGHUA Hospital Shanghai University of Traditional Chinese Medicine.

### Cell viability

Cell viability was determined by using cell-counting kit 8. After the cells were seeded into a 96-well microplate and treated, cell-counting kit 8 reagent was added as the manufacturer instructed. After incubating for 2 hours, the plate was read at a wavelength of 460 nm.

### Biochemical assays

After the treatment, lipid ROS was measured by using C11-BODIPY dye according to the instruction (Thermo Fisher Scientific, Waltham, MA, USA). In brief, the cells were incubated with C11-BODIPY dye (5 μM) for 30 mins followed by washing with phosphate buffered saline solution twice. The plate was then read at a wavelength of 510 nm. To measure the iron concentrations, QuantiChrom Iron Assay Kit was used after treatment, according to the manufacturer's document (Bioassay Systems, Hayward, CA). The plate was read at a wavelength of 460 nm.

### Construction of cell line

FSP1 shRNA (target sequence: CGGGCAAGTTTAATGAGGTTT) and negative control shControl were obtained from Sigma-Aldrich (St. Louis, MO). After the shRNA sequence was inserted into pLKO.1 backbone. The plasmids were used to generate the lentivirus particles. The cells were then transfected by the lentivirus particles followed by the selection of Puromycin. To construct FSP1 overexpressing cell lines, pcDNA3.0 was used to insert the FSP1 CDS sequences. The PC3 cell line was then transfected with the engineered plasmids.

### qPCR

Total RNAs were extracted from the Taqman MicroRNA Assay Kits (Thermo Fisher Scientific). Sequences of the primers were described as follows: FSP1 forward: 5'- AGA CAG GGT TCG CCA AAA AGA-3', and reverse: 5'-CAG GTC TAT CCC CAC TAC TAG C-3'; SLC7A11 forward: 5'-TCT CCA AAG GAG GTT ACC TGC-3', and reverse: 5'- AGA CTC CCC TCA GTA AAG TGA C-3'; SLC3A2 forward: 5'-TGA ATG AGT TAG AGC CCG AGA-3', and reverse: 5'-GTC TTC CGC CAC CTT GAT CTT-3'; GPX4 forward: 5'- GAG GCA AGA CCG AAG TAA ACT AC-3', and reverse: 5'-CCG AAC TGG TTA CAC GGG AA-3'; GADPH forward: 5'-TGT GGG CAT CAA TGG ATT TGG-3', and reverse: 5'- ACA CCA TGT ATT CCG GGT CAA T-3'. Reverse transcription assay was then performed as described follows: denaturing at 95˚C for 5 mins, followed by 95˚C (5 seconds) and 60˚C (1 min) with 40 cycles. 2^-ΔΔCq^ was used to calculate the levels of each target and *GADPH* was used as an internal control.

### Western blotting

Proteins were extracted from the cells or the tissues by using a lysis buffer. After protein quantification, an equal amount of protein (20 μg) was loaded into 10% SDS-PAGE gel followed by the membrane transfer. After blocking with 5% non-fat milk, the membrane was then blotted with primary antibodies against GADPH (1:3000), FSP1(1:1000), SLC7A11(1:1000), SLC3A2 (1:1000), and GPX4 (1:800) at 4˚C overnight. After blotting with the secondary antibodies, the membrane was visualized by using Chemiluminescence Kit (MilliporeSigma, Burlington, MA).

### Statistical analysis

The data were displayed as the means ± standard deviation (SD). GraphPad Prism7 was used to compare the significant difference among groups by using t-test, one-way or two-way ANOVA test.

## Results

### QLD suppressed the levels of FSP1 in patients with CRPC

**Figure 1:**
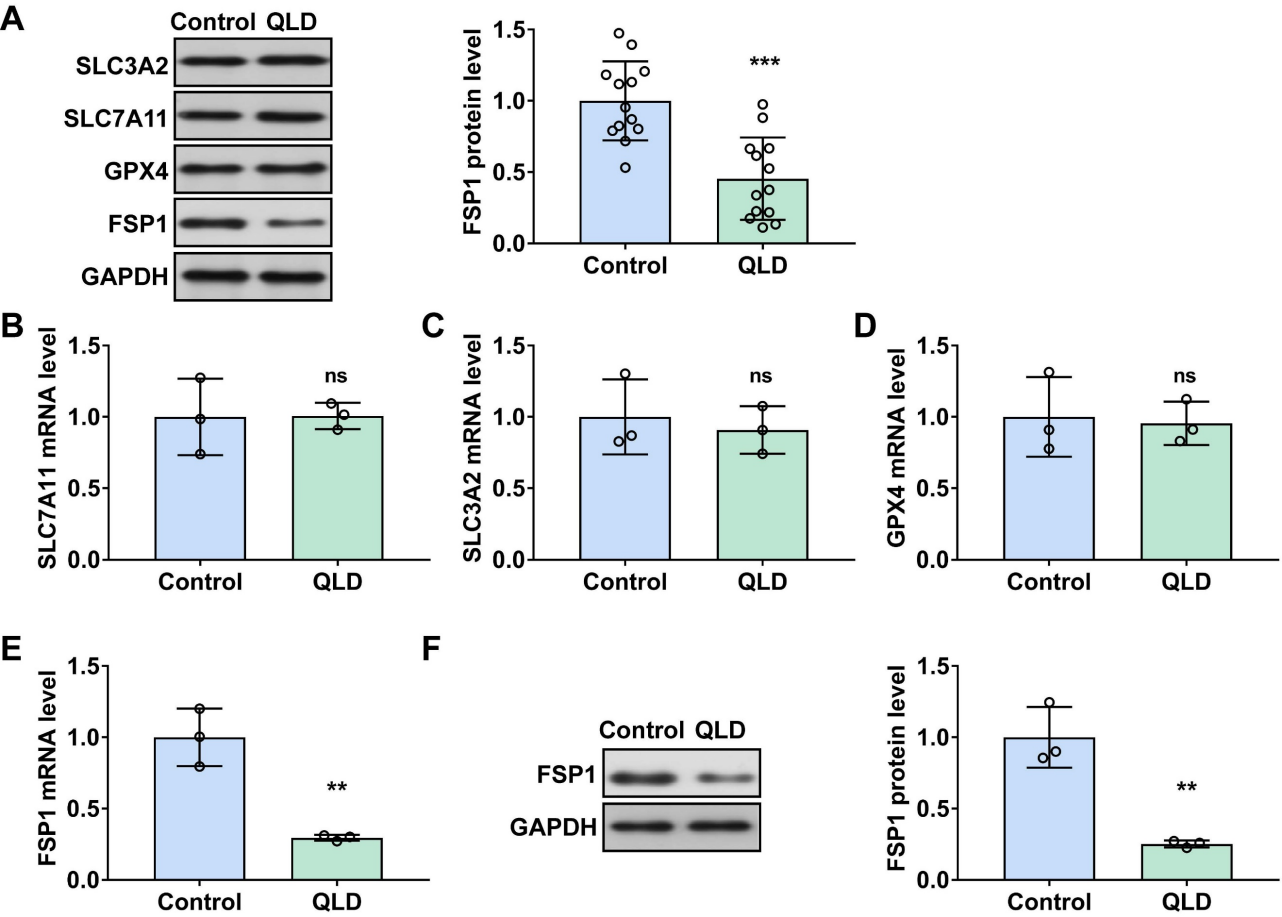
** QLD suppressed the levels of FSP1 in patients with CRPC.** (A) The protein expressions of ferroptosis-related markers including SLC7A11, SLC3A2, GPX4, and FSP1 were determined by using Western blotting in the CRPC patients pre- and post-QLD treatment (n= 13). (B-E) The mRNA levels of SLC7A11, SLC3A2, GPX4, and FSP1 were detected by qRT-PCR in the PC3 cells that were treated with 2% control serum and 2% QLD serum for 48h. (F) The protein expressions of FSP1 were determined by Western blotting in the PC3 cells that were treated with 2% control serum and 2% QLD-containing serum for 48h (n = 3). The significant difference was determined by t-test. ** *p* < 0.01, *** *p* < 0.001, ns indicates no significant difference.

Before and after the treatment of QLD, we measured several ferroptosis-related biomarkers (SLC7A11, SLC3A2, GPX4, and FSP1) in tumor tissues from the patients with CRPC. The clinic parameters showed the efficacy of QLD on CRPC patients (Table S1). Interestingly, we found that FSP1 was significantly decreased in the QLD-treated patients (Fig. 1A). However, no significant changes in other ferroptosis-related biomarkers including SLC7A11, SLC3A2, and GPX4 were observed in the patients before and after treatment of QLD. Consistently, in PC3 cells incubated with 2% QLD-containing serum, we found that FSP1 was significantly decreased (Fig. 1E and F), whereas no significant changes in ferroptosis-related biomarkers including SLC7A11, SLC3A2, and GPX4 were observed (Fig. 1B-D), as compared to the cells incubated with 2% control serum.

### QLD promoted ferroptosis in the PC3 cells

**Figure 2:**
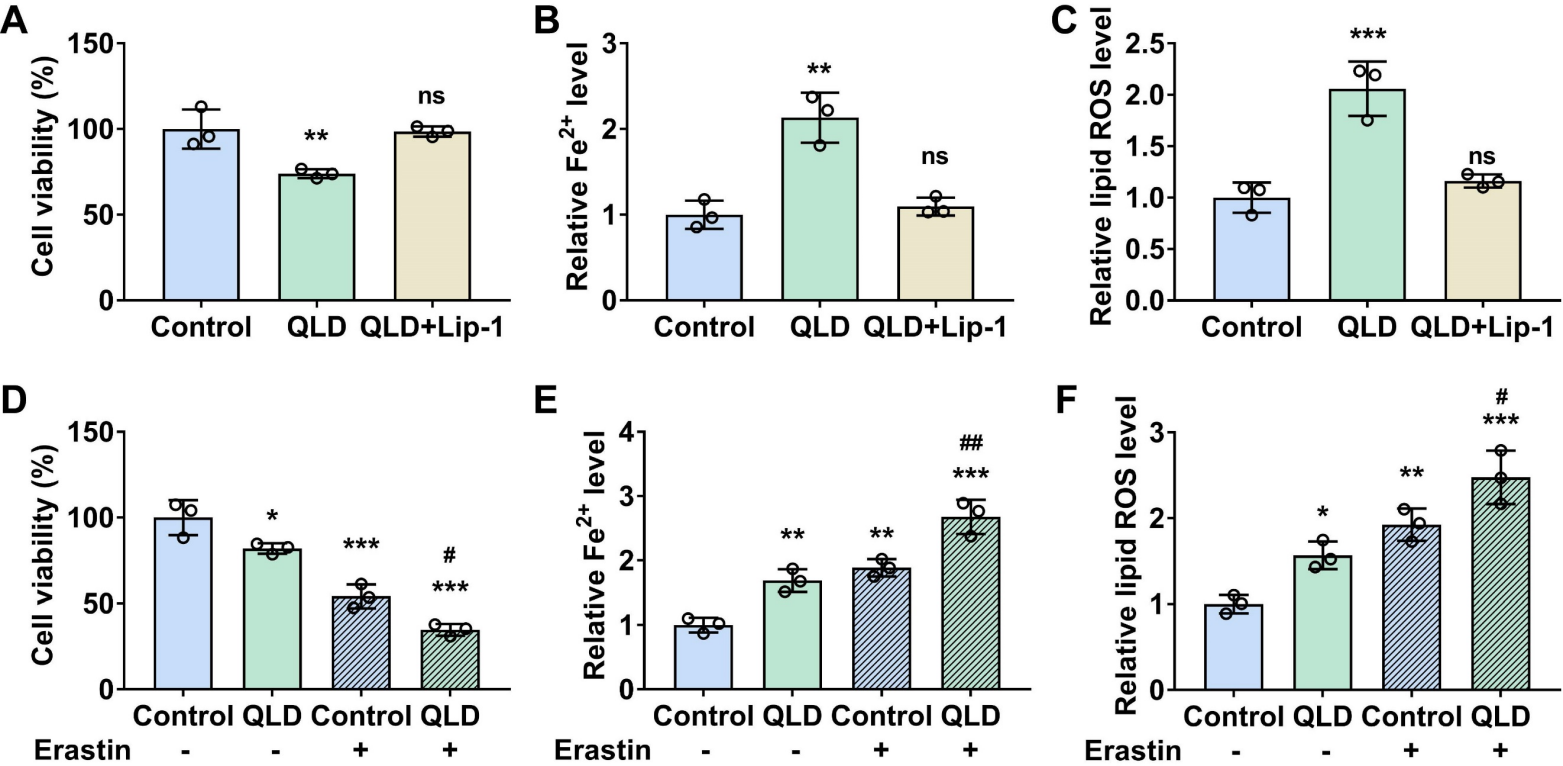
** QLD promoted ferroptosis in the PC3 cells.** PC3 cells were treated with 2% QLD serum with or without Lip-1 (20 μM) for 48 h. PC3 cells that were treated with 2% control serum for 48 h were used as the control. The cell viability (A), Fe^2+^ (B), and Lipid ROS (C) levels were then measured (n = 3). (D-F) PC3 cells were treated with 2% control serum or 2% QLD serum with or without Erastin (1 μM) for 24h. The cell viability (D), Fe^2+^ (E), and Lipid ROS (F) levels were measured (n = 3). The significant difference was determined by using one-way ANOVA. * *p* < 0.05, ** *p* < 0.01, *** *p* < 0.001 compared with control group. # *p*<0.05, ## *p*<0.01 compared with Control plus Erastin group. ns indicates no significant difference.

Next, we investigated whether QLD could affect cell ferroptosis. Our results showed that cell viability was decreased (Fig. 2A), whereas Fe^2+^ and lipid ROS levels were significantly increased in the PC3 cells incubated with 2% QLD containing serum (Fig. 2B and C), as compared to those cells treated with 2% control serum. In addition, we also evaluated the effects of QLD on ferroptosis using C11-BODIPY fluorescence staining. Consistently, the cell images and statistical analysis indicate that QiLing Decoction induces ferroptosis in PC3 cells (Fig. S1A). Moreover, we treated normal prostatic epithelial (RWPE-1) cells with QLD and found that it did not cause a decrease in cell viability or induce ferroptosis (Fig. S1B-1D). However, QLD was able to induce ferroptosis in another CRPC cell line, DU145 cells (Fig. S1E-1G). These results suggest that QLD may be capable of specifically inducing ferroptosis in PCa cells, while not inducing ferroptosis in prostate epithelial cells, demonstrating the specificity of QLD in inducing ferroptosis. Interestingly, in the presence of Lip (ferroptosis inhibitor), cell viability was increased (Fig. 2A), whereas Fe^2+^ and lipid ROS levels were significantly decreased in the PC3 cells incubated with 2% QLD-containing serum (Fig. 2B and C). These results suggested that ferroptosis inhibitor suppressed PC3 cells ferroptosis induced by QLD. Additionally, we also determined the effects of QLD on ferroptosis in the presence of a ferroptosis inducer. Our results revealed that QLD promoted PC3 cell ferroptosis induced by erastin, as supported by treatment of QLD resulted in a further decrease the cell viability (Fig. 2D), and an elevation of Fe^2+^ and lipid ROS levels of PC3 cells induced by erastin (Fig. 2E and F).

### QLD promoted ferroptosis in the PC3 cells by inhibiting FSP1

**Figure 3:**
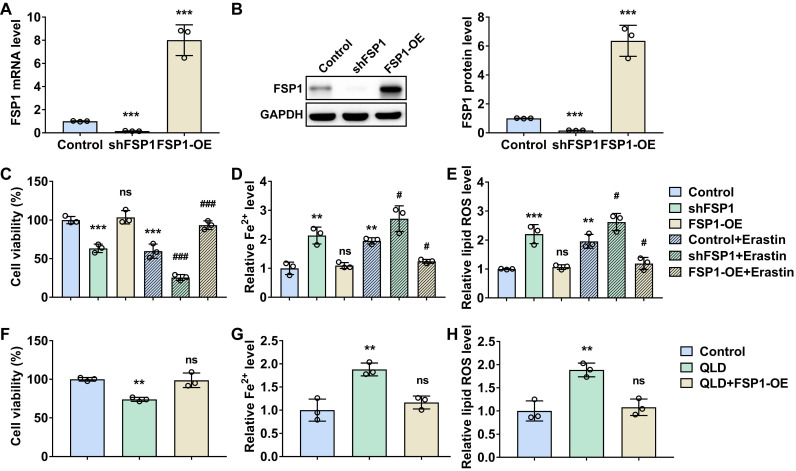
** QLD promoted ferroptosis in the PC3 cells by inhibiting FSP1.** (A-B) PC3 cells were transfected with control plasmids, shFSP1 or FSP1-overexpressing (OE) plasmids for 48h. The mRNA and protein levels of FSP1 were detected by using qRT-PCR and Western blotting, respectively (n = 3). (C-E) After the transfection of 24 h, PC3 cells were then treated with or without Erastin (1 μM) for another 24h. Then the cell viability, Fe^2+^, and lipid ROS were measured by using commercialized kits (n = 3). (F-H) PC3 cells that were treated with 2% control serum or 2% QLD serum were transfected with the FSP1-OE plasmids for 48h. Then the cell viability (F), Fe^2+^ (G), and Lipid ROS (H) levels were measured (n = 3). The significant difference was determined by using one-way ANOVA. ** *p*<0.01, *** *p*<0.001 compared with control group. # p<0.05, ### p<0.001 compared with control plus Erastin group. ns indicates no significant difference.

We then determined whether the regulatory effects of QLD on ferroptosis were associated with FSP1. First, we successfully established FSP1 knockdown and overexpressing cell lines, as supported by the changes in FSP in these cell lines (Fig. 3A and B). Next, we determined cell viability, Fe^2+^, and lipid ROS levels in these cell lines with or without erastin. Interestingly, we found that FSP1 knockdown led to a decrease in cell viability, and an increase in Fe^2+^, and lipid ROS levels, whereas FSP1 overexpression recovered cell viability and suppressed Fe^2+^ and lipid ROS levels (Fig. 3C-F). In addition, erastin induced cell ferroptosis, whereas FSP1 knockdown further accelerated cell ferroptosis, as suggested by a decrease in cell viability, and an increase in Fe^2+^, and lipid ROS levels in erastin-induced cells. However, FSP1 overexpression recovered cell viability and suppressed Fe^2+^ and lipid ROS levels in erastin-induced cells (Fig. 3C-F). Next, we incubated FSP1 overexpression cell lines with 2% QLD-containing serum. We found that cell viability was increased, and Fe^2+^ and lipid ROS levels were suppressed in those FSP1 overexpression cell lines incubated with 2% QLD-containing serum, as compared to PC3 cells treated with 2% QLD-containing serum (Fig. 3G-H).

### QLD inhibited the tumor growth by inhibiting FSP1

**Figure 4:**
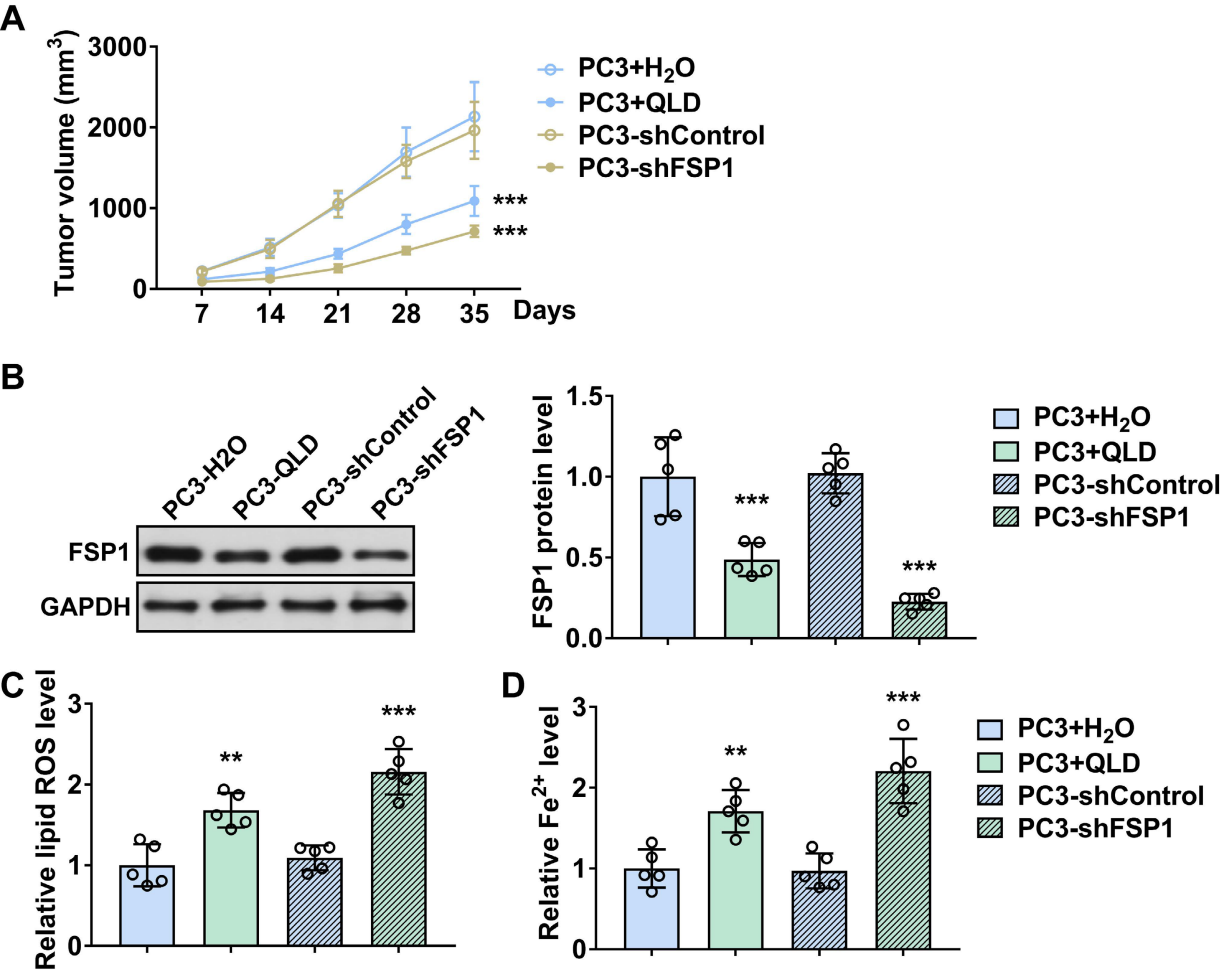
** QLD inhibited tumor growth by inhibiting FSP1 *in vivo*.** (A) Tumor volumes were measured every seven days (n = 5). The significant difference was determined by two-way ANOVA. (B) on day 35, tumor tissues were collected and the protein expressions of FSP1 were determined by using Western blotting. The levels of Fe^2+^ (C) and lipid ROS (D) in tumor tissue were also detected (n = 5). The significant difference was determined by using one-way ANOVA. ** *p* < 0.01, *** *p* < 0.001 compared with corresponding control group. ns indicates no significant difference.

We further investigated the effects of QLD on tumor growth. We found that tumor volume was reduced in QLD-treated mice as compared to the control group (Fig. 4A). Similarly, mice xenografted with shFSP1-transfected cells also reduced tumor volume as compared to the control group (Fig. 4A). On day 35, we collected tumor tissue and detected the levels of FSP1. Interestingly, we found that FSP1 was suppressed in tumor tissues from the QLD-treated mice and mice xenografted with shFSP1-transfected cells (Fig. 4B). Moreover, we found that relative lipid ROS and Fe^2+^ levels were significantly increased in QLD-treated and shFSP1-transfected groups (Fig. 4C and D).

### QLD increased the sensitivity of PC3-AbiR cells to abiraterone by inhibiting FSP1

**Figure 5:**
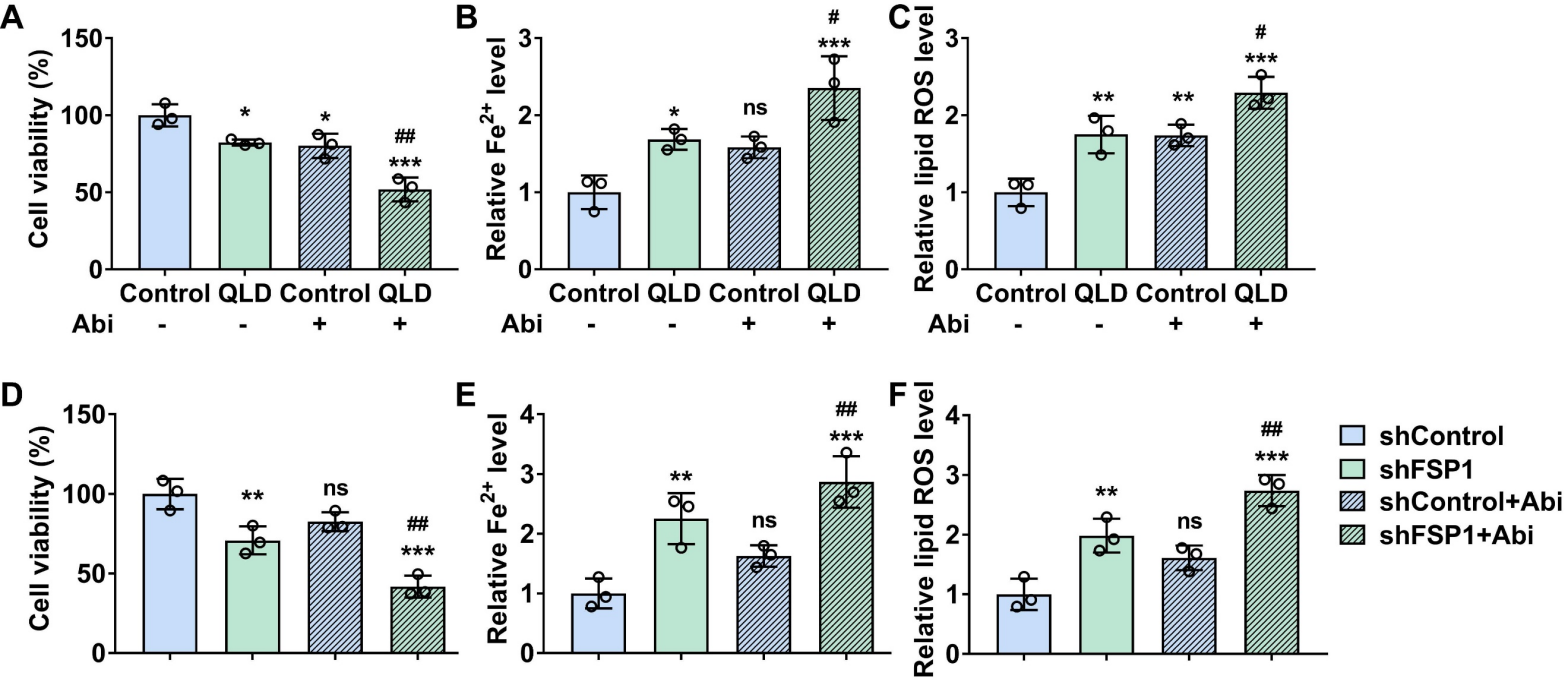
** QLD increased the sensitivity of PC3-AbiR cells to abiraterone by inhibiting FSP1*.*** PC3-AbiR cells were incubated with 2% control serum or 2% QLD serum with or without abiraterone (20 μM) for 48h. Next, the cell viability (A), Fe^2+^ (B), and lipid ROS (C) were measured (n = 3). (D-F) PC3-AbiR cells were transfected with shControl or shFSP1 plasmids with or without abiraterone (20 μM) for 48h. After that, the cell viability (D), Fe^2+^ (E), and Lipid ROS (F) levels were measured (n = 3). The significant difference was determined by using one-way ANOVA. * *p* < 0.05, ** *p* < 0.01, *** *p* < 0.001 compared with Control group. # *p* < 0.05, ## *p* < 0.01 compared with Control plus Abi group. ns indicates no significant difference.

We explored whether QLD affected the sensitivity of PC3-AbiR cells to Abi. We found that cell viability of PC3-AbiR cells induced by Abi (20 μM) was further decreased in the cells treated with 2% QLD containing serum (Fig. 5A). Furthermore, both relative lipid ROS and Fe^2+^ levels were significantly increased in the Abi-treated cells in the presence of 2%-QLD containing serum (Fig. 5B and C). We then investigated whether the effects of QLD on the sensitivity of PC3-AbiR cells to Abi were associated with FSP1. Our results showed that cell viability was decreased in the shFSP1-transfected cells treated with Abi, as compared to shControl-transfected cells treated with Abi (Fig. 5D). Moreover, both relative lipid ROS and Fe^2+^ levels were significantly increased in the shFSP1-transfected cells treated with Abi, as compared to shControl-transfected cells treated with Abi (Fig. 5E and F).

### QLD increased the sensitivity of PC3-AbiR tumor to Abi by inhibiting FSP1

**Figure 6:**
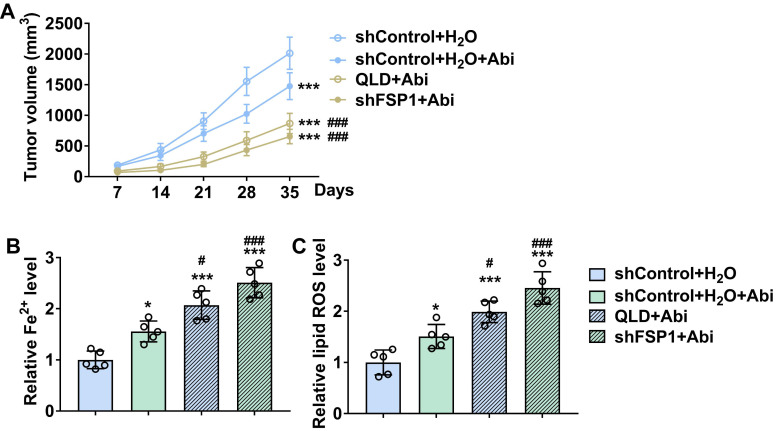
** QLD increased the sensitivity of the PC3-AbiR tumor to abiraterone by inhibiting FSP1.** (A) Tumor volumes were measured every seven days (n = 5). The significant difference was determined by using two-way ANOVA. *** *p* < 0.001 compared with shControl plus H_2_O group. ### *p* < 0.001 compared with shControl plus H_2_O plus Abi group. (B-C) On day 35, tumor tissues were collected, and the levels of Fe^2+^ (B) and lipid ROS (C) in tumor tissue were detected (n = 5). The significant difference was determined by using one-way ANOVA. * *p* < 0.05, *** *p* < 0.001 compared with shControl plus H_2_O group. # *p* < 0.05, ### *p* < 0.001 compared with shControl plus H_2_O plus Abi group. ns indicates no significant difference.

We further conducted the xenograft tumor model to verify the roles of QLD in regulating the sensitivity of the PC3-AbiR tumor to Abi. We found that treatment of Abi significantly reduced tumor volume in PC3-AbiR xenograft tumors (Fig. 6A). Additionally, treatment of Abi further reduced tumor volume in the mice subcutaneously injected with QLD-treated or shFSP1-transfected PC3-AbiR cells (Fig. 6A). Interestingly, our results revealed that treatment of Abi increased lipid ROS and Fe^2+^ levels in the tumor tissues from mice subcutaneously injected with QLD-treated or shFSP1-transfected PC3-AbiR cells (Fig. 6B and C).

## Discussion

Many studies have reported that ferroptosis is associated with the initiation and progression of prostate cancer 11,17,12,18. The inhibitory effects of ferroptosis against prostate cancer are associated with a series of cellular events (e.g., autophagy, apoptosis) and signaling proteins (e.g., p53, NF-E2-related factor 2) 17. Interestingly, several studies suggested that inducing ferroptosis is a novel therapeutic approach against advanced prostate cancer, such as CRPC 12,18. For instance, Yang and colleagues reported that the combination of erastin, ferroptosis inducer, with docetaxel, enhances the inhibitory effects of docetaxel against CRPC tumor growth by the regulation of androgen receptor 19. Another study reported that inducing ferroptosis can increase the sensitivities of prostate cancer cells to an androgen receptor inhibitor, enzalutamide 20. These results suggested that inducing ferroptosis might be one option for drug-resistant prostate cancer therapy.

QLD is widely used for CRPC therapy in many clinical settings in China 16,15. The combination of QLD with docetaxel improves the therapeutic effectiveness of docetaxel against CRPC by enhancing cancer cells' sensitivities to docetaxel cytotoxicity 14. Another study revealed that the application of QLD enhances the therapeutic effects of abiraterone against CRPC, in part, by the regulation of autophagy 16. These results suggested that QLD may contribute to overcoming drug resistance in advanced prostate cancer therapy. However, it is still unclear its underlying mechanisms. Herein, this study aims to explore whether the effects of QLD on CRPC were associated with ferroptosis.

First of all, we investigated the effects of QLD on the ferroptosis-related biomarkers in CRPC tissues. The occurrence and progression of ferroptosis are involved in several signaling proteins including GPX 4, SLC7A11, SLC3A2, and FSP1 21. GPX 4 is a master regulator for reducing complex hydroperoxides and disrupting lipid peroxidation 22. FSP1 is a ferroptosis suppressor that is mediated by ubiquinone, which combats the lipid peroxidation induced by GPX4 23. Amino acid transporters including SLC7A11 and SLC3A2 regulate cystine uptake and the biosynthesis of glutathione 24. Interestingly, in clinical specimens, we observed that FSP1 was significantly decreased in the tumor tissues from QLD-treated patients. Consistently, in PC3 cells incubated with 2% QLD-containing serum, a decrease of FSP1 was also observed. These results suggested that treatment of QLD inhibited FSP1, a ferroptosis suppressor, in advanced prostate cancer. While the findings of this study are promising, a limitation is the relatively small sample size (n=13). This is part due to the limited availability of patient samples in our department. Future studies with larger sample sizes are warranted to further confirm our observations.

Second, we explored whether QLD affected ferroptosis in prostate cancer cells. Ferroptosis is characterized by the accumulation of Fe^2+^, and lipid peroxides 9,8. In this study, we determined cell viability, relative Fe^2+^ and lipid ROS. Interestingly, we found that PC3 cells that were incubated with 2% QLD-containing serum displayed a decrease in cell viability and an increase in Fe^2+^ and lipid ROS levels. Additionally, we found that the ferroptosis inhibitor reversed the changes caused by QLD, as supported by an increase in cell viability and a decrease in Fe^2+^ and lipid ROS levels. However, in the presence of the ferroptosis inducer, cell viability was increased, whereas Fe^2+^ and lipid ROS levels were significantly increased in the PC3 cells incubated with 2% QLD-containing serum. These results suggested that QLD promoted ferroptosis in prostate cancer cells.

Third, we explored whether the effects of QLD on ferroptosis were associated with FSP1 in prostate cancer. Our *in vitro* and *in vivo* results suggested that the regulatory effects of QLD on ferroptosis were associated with FSP1. FSP1, known as a ferroptosis suppressor, is independent of glutathione, which cooperates with GPX4 to inhibit ferroptosis 25. As expected, knockdown FSP1 promoted ferroptosis, whereas its overexpression suppressed ferroptosis. Interestingly, ferroptosis that were induced by QLD were suppressed in those FSP1 overexpression cell lines. *In vivo*, FSP1 was suppressed in tumor tissues from the QLD-treated mice. These results suggested that the effects of QLD on ferroptosis in prostate cancer were associated with its regulatory effects against FSP1.

Drug resistance is still one of the major challenges in the treatment of CRPC 26. For instance, Abi is widely used for those patients with advanced prostate cancer who do not respond to other hormone therapies 27. However, Abi resistance is still observed in some portions of the patients 28. In this study, we further investigated the effectiveness of QLD on the Abi resistance in CRPC and whether the underlying mechanisms were associated with ferroptosis. Interestingly, we observed that QLD affected the sensitivity of PC3-AbiR cells to Abi in vitro. Consistently, treatment of Abi significantly reduced tumor volume in the mice that were subcutaneously injected with QLD-treated cells. These results suggested that QLD increased the sensitivity of prostate cancer cells to Abi therapy. Our further results revealed that ferroptosis was enhanced in the shFSP1-transfected cells treated with Abi, as compared to shControl-transfected cells treated with Abi. Taken together, these results suggested that the regulatory effects of QLD on Abi resistance were associated with its inhibitory effects against FSP1.

In this study, we present a novel perspective on the mechanism of drug resistance in prostate cancer and propose new treatment strategies for clinical prostate cancer therapy based on cell ferroptosis. Moreover, we report, for the first time, the regulatory effect of QLD on ferroptosis in prostate cancer cells and its physiological function in abiraterone resistance. Additionally, we uncover the regulatory role of FSP1 in abiraterone resistance of prostate cancer, which offers new insights for overcoming prostate cancer drug resistance.

## Conclusion

Clinical specimens showed that treatment of QLD inhibited FSP1 in patients with CRPC. In vitro, QLD promoted cell ferroptosis in PC3 cells. The regulatory effects of QLD on ferroptosis were associated with its inhibitory effects against FSP1, as supported by the fact that QLD inhibited PC3 cancer cell proliferation and tumor growth by inhibiting FSP1. Moreover, QLD also increased the sensitivity of PC3-AbiR cells to Abi by inhibiting FSP1. These results suggested that QLD promoted ferroptosis and the sensitivity of PC3-AbiR cells to abiraterone in part by inhibiting FSP1.

## Supplementary Material

Supplementary figure and table.
