# Supplementary material for: QiLing Decoction promotes ferroptosis of castration-resistant prostate cancer cells by inhibiting FSP1 in vitro and in vivo
Source: J Cancer. 2023 Jul 16;14(12):2236–45. doi: 10.7150/jca.84363 (PMC10414048; doi:10.7150/jca.84363)
Supplement: Supplementary file 1 — Supplementary figure and table. [file jcav14p2236s1.pdf]

Supplementary Materials

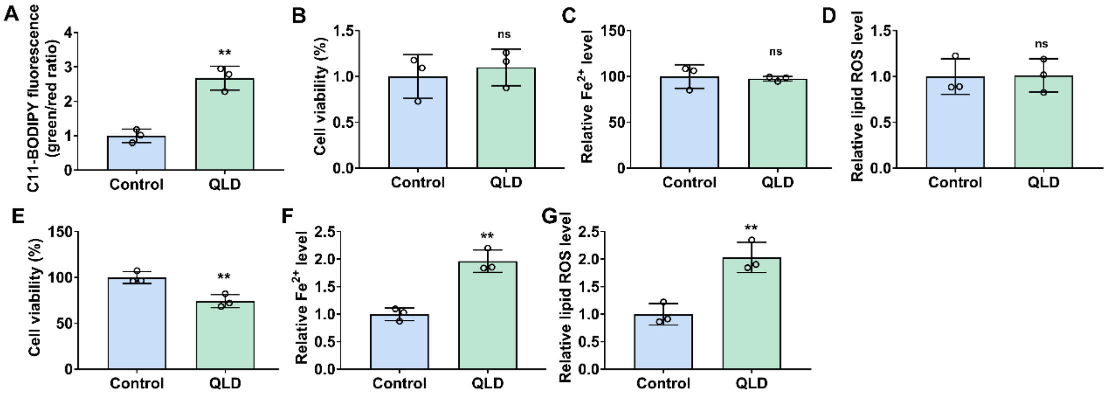

Figure S1. QLD promoted the ferroptosis in prostate cancer cells but not normal prostate cells. PC3 cells were treated with either 2% control serum or 2% QLD serum for 12 hours, and the oxidized/non-oxidized lipids ratio was calculated using C11-BODIPY staining (A). Additionally, normal human prostatic epithelial cells (RWPE-1) and DU145 cells were treated with 2% control serum or 2% QLD serum for 48 hours, and the cell viability (B, E), Fe2+ (C, F), and lipid ROS (D, G) levels were measured (N = 3, t-test, ** $p < 0.01$, ns: no significant difference).

Table S1. Clinic parameters before and after QLD treatment.

	before QLD (n=13)	after QLD (n=13)	*p* value
prostate volume (cm^3)	40.45±12.31	36.96±12.17	0.47
PSA (mg/L)	11.26±1.53	9.31±1.11	0.0011
Maximum urinary flow rate (mL/s)	10.34±0.94	11.53±0.84	0.0023
KPS	57.72±5.68	65.77±7.82	0.0062
VAS	4.27±2.12	1.88±1.27	0.0019

PSA, prostate-specific antigen

KPS, KPS life quality score

VAS, Visual analogue scale, a pain score system

The data were displayed as the means ± standard deviation (SD).

p value was analyzed by using t-test.
